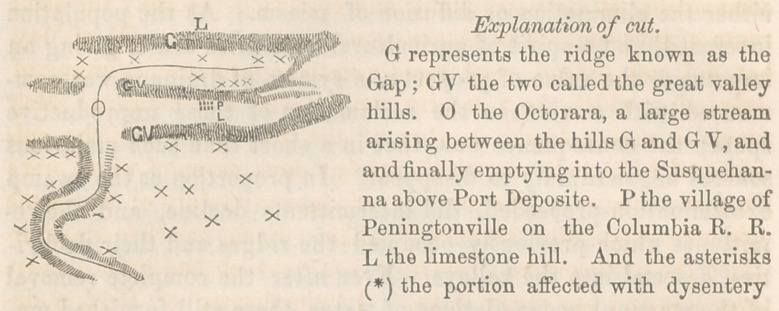# Medical Topography, with Observations on the Epidemic Dysentery of Lancaster County

**Published:** 1852-04

**Authors:** D. H. Agnew

**Affiliations:** Philadelphia


### MEDICAL EXAMINER,

AND

### RECORD OF MEDICAL SCIENCE.

NEW SERIES.—NO. LXXXVIII.—APRIL, 1852.

### ORIGINAL COMMUNICATIONS.

*Medical Topography, with Observations on the Epidemic Dysentery of Lancaster County. With a cut.* By D. H. AGNEW, M. D., Philadelphia.

To prosecute our inquiries into the regions of epidemic causation with any prospect of success, the medical topography of a country should be made the first matter of consideration. Every physician who has devoted a few years to the active duties of his profession, will have discovered a tendency in certain diseases to apportion particular districts to their exclusive domination. The expression of such well marked local predilections, suggests at once the existence of some local principle favorable to their production; while their periodical visitations no less strongly prove the necessity for certain meteorological conditions to quicken them into activity. Now although we may not be able, by our present knowledge and means of observation, to isolate this subtle principle, or compel a respondence even by the most delicate reagents; still, with the overwhelming evidence before us, we are bound to admit its existence, and regret only the imperfection of our analysis. When we glance at the wonderful results obtained by physicists in the various departments of physical science, not only in their investigations of the more gross forms of material substances, but in the appreciation and mathematical measurements of even those infinitessimal undulations of ether, which give rise to certain modifications of light, we have the greatest encouragement to believe that even the tenuous and highly sublimated principle of contagion will yet succumb to the triumphs of modern chemistry. If it be true then that disease can be localised, and indicated by geographical lines, undoubtedly a study of the physical peculiarities of such regions would be calculated to throw much valuable light upon their etiology. There is a prevailing expression among medical men, and obvious even to the eyes of the vulgar, that diseases have changed, and the natural interrogatory arises, to what are we to attribute this change? Judging from observations made in my own district, and which are submitted in a brief and cursory manner, I am forced to believe the explanation resides in the physico-moral history of a neighborhood. In the locality referred to, many years ago, intermittent and remittent fevers were the prominent and prevailing affections. The points which they respectively occupied, exhibit in a striking degree the dependence of each upon the same morbific cause, and it seems exceedingly probable that the degree of miasmatic concentration alone determines the type of fever. This may be modified either in consequence of the limited supply of productive materials, or locality. In elucidating the above, I will state that there existed in this region several quite extensive marshes. On the low grounds or valleys, contiguous to these sources of pestiferous emanations, intermittent cases greatly preponderate ; while on the more elevated grounds, remittent fevers alone exist, showing a modification in its sensible effects either by admixture or altitude. The influence of locality was most decidedly expressed, in another portion of the same district, where those families residing on the east side of a marshy portion of ground were invariably affected with ague, whilst the inhabitants on the west, many of whose houses were on its very verge, entirely escaped. These facts I attributed to the currents of air. During clear weather the north-western winds prevail, wafting these paludal exhalations over the dwellings of the eastern residents. On the other hand, when the contrary or eastern winds existed, they were generally accompanied by rain, and those meteorological conditions of air, light and heat unfavorable to either the elimination or diffusion of miasm. As the population increased, and a spirit of agricultural enterprise arose, giving an impetus to the value of property, a system of drainage was commenced with a view to the reclamation of these unproductive spots; the consequence was, that in a short time such malarious sources began rapidly to disappear. In proportion as the swamp extermination proceeded, did intermittents decline, and the remittents which previously occupied the ridges and their declivities, descend into the valleys. Even after the complete removal of the stagnant accumulations of water, these still furnished materials for the production of a certain amount of malaria, in consequence of the streams which flowed through them being narrow and tortuous, and thus rendered inadequate to conduct away any unusual accumulations of water which occurred during violent rains. At such periods large quantities of decomposed organic matter were carried down by the swollen tributaries from the hill sides; and the main channel overflowing its banks, a rich deposite was left upon its subsidence, to be acted upon by solar heat. In the mean time the profits arising from grazing cattle began to attract the attention of agriculturists; the meadow lands, from the character of their grasses being eminently adapted for this purpose, assumed a new degree of importance, and measures were put at once into operation, to render them as valuable as possible by widening and straightening their channels. By this improvement they seldom overflow; and for the last few years, remittent fever has almost entirely disappeared. The present affections which, from their numerical strength, rise to the rank of prevailing or epidemic diseases, are typhoid fever, and dysentery. The exanthematous class, originating as they do, in a specific poison, and communicable by contact, of course it is not presumed to make them dependent upon any local influences of a physical character. The employments, and alimentation of our race at the present day, are calculated to develope the nervous unduly over the muscular system, and this very disproportionate distribution of nutrition may be rationally invoked to explain the prevailing typhoid complexion of our fevers. The partiality manifest by colitis for those portions of my district marked upon the cut, I am disposed to attribute to geological causes.

*Explanation of cut.*

G represents the ridge known as the Gap; GV the two called the great valley hills. O the Octorara, a large stream arising between the hills G and G V, and and finally emptying into the Susquehanna above Port Deposite. P the village of Peningtonville on the Columbia R. R. L the limestone hill. And the asterisks (*) the portion affected with dysentery

The country represented here embraces a portion of Lancaster and Chester counties. It will be perceived that there are three chains of ridges running almost parallel with one another, and dividing the intermediate country into three distinct valleys. two of which, the most northern and southern, known as the Pequea and the Great valleys, are of very considerable extent, while the intermediate one, in consequence of spurs being sent off from the adjoining hills, is converted into a basin of a few miles. Within the northern and middle ridges, the tributary sources of the Octorara arise, the main stream of which is seen taking a course west and south, skirted with high hills on either side. The vallies, north and south, are limestone; and reposing against the southern declivity of the middle ridge, is seen the village of Peningtonville on the edge of the Great valley. In all the epidemics which I have witnessed of the disease, this village invariably furnished the first cases. In 1842 the disease commenced about the first of August. The month of July previous contained fifteen days of unfair weather, seven of which were showery, and from the 27th to the 31st the thermometer did not fall below 95° during the day. For a short time previous, various intestinal disorders presented themselves, foreshadowing its approach. The first cases occurred at Peningtonville, and from thence it extended north, invading all that portion of the neighborhood marked by the asterisks, to the Pequea valley, (a limestone district) where it was arrested by this apparent cordon sanitaire. Striding over the Great valley immediately in front of the village, (also limestone,) it appeared immediately on its southern verge, and travelled in that direction far beyond the limits of my district. In 1844 it again appeared as an epidemic, commencing at the same point, and observing precisely the same route of invasion. The first cases occurred about the 5th of May, but did not extend very rapidly until toward the beginning of July, after which they greatly multiplied. The month of May previous, was analogous to that of the July preceding the epidemic of 1842, in having sixteen unfair days, seven of which were likewise showery. The months of June and July of the same epidemic were also singularly alike. Each had five days in which the average temperature was 97°; each had light cloudy, three wet and two foggy days. In the disease of 1842, a decrease in the number of cases occurred after the 13th of September; and on the 23d was brought to a sudden arrest by a brisk frost; no new cases took place after that date, and such as were laboring under an attack, commenced a rapid convalescence. In the one of 1844, the declension was very gradual and straggling; cases were seen until the weather became decidedly cool. Now, in neither of these visitations of the disease, did I have a single case occurring in the families of those residing in the valleys and using limestone water, and it is to this cause that I am disposed to attribute their exemption. In what manner limewater acts as a prophylactic is not easily explained; for with many, water thus impregnated deranges the bowels very considerably. The power of lime as a disinfecting and corrective agent, is generally admitted, and it may perhaps exert some alterative action in the system, rendering it insusceptible to morbid impressions of this character.

Why the colon should be the seat of disease in preference to any other portion of the intestinal canal, is another question of some obscurity. May it not be accounted for on the ground of difference of function? The small intestines are more exclusively the organs of absorption. The chyme being a bland unirritating mixture, rather protects the mucous coat of the bowel from injurious impressions than otherwise; while the large intestines being the receptacle of the excrementitious matter of alimentary substances (unfit for the purposes of nutrition) and the various secretions of intestinal glandulæ, are exposed to much greater mechanical irritation, in addition to which the developement of gases arising from a degree of decomposition going on, may add to the efficiency of this cause.

The disease, as it prevailed in Lancaster county, commenced with the ordinary griping pains, frequent inclinations to evacuate the bowels, attended with straining, tender belly, and the discharge of a small portion of mucus of the consistence of jelly, and generally intermixed with streaks of blood. Others passed a thin brown fluid containing long, tough, and tenacious shreds, similar to the scrapings of guts. The tongue was sometimes loaded with a heavy brown coat; at others, dry and red; the heat of skin somewhat elevated above the natural standard, and in about 24 hours subsequent to such evidences of indisposition, a symptomatic fever set in. In those cases where but little blood appeared in the evacuations, the sufferings of the patients were much aggravated, and the tenesmus more annoying, amounting to tormina, and such appeared to indicate a higher degree of inflammation, and demanded the employment of active antiphlogistic remedies. When the bloody discharges preponderated, my impression was that the congestion and inflammation were, to a certain extent, relieved by this kind of local depletion.

*Treatment.*—On this subject, much diversity of opinion exists. The use of the lancet is viewed by many with great distrust, and not unattended with danger, while that of mercury as downright injurious, and deserving utter proscription. Some admit the propriety of occasionally administering a mercurial, merely with a view of stimulating the hepatic apparatus, but rely mainly upon opium for subduing the intestinal disease. While others discard, in the main, all the above, and treat every case by a thorough course of purgatives, either with a view to relieve the engorged state of the mucous membrane, or dislodge accumulations of hardened scybala, visible, in most instances, only to the eye of faith. The great reason why the abstraction of blood is not deemed requisite in this affection, arises, no doubt, from an under estimate of the grade of inflammatory action, and this is the rock upon which many an unfortunate patient is sacrificed. The truth is, the disease is one of intense phlogosis, otherwise it would be impossible to explain the organizations and extensive structural alterations revealed to the pathologist. The pulse, though a pretty correct guide in most diseases, fails in many cases of dysentery to indicate the degree of danger ; and hence, if we expect to find the full bounding vessel as in pleuritis, or the hard contracted one characteristic of peritonitis, we shall be very greatly disappointed. Again, if the administration of mercury be practised only under the idea that the liver is permanently affected in all cases of colitis, we think such will be doomed to disappointment, as the investigations of the ablest pathologists fail to establish hepatic complications, other than accidental to the general disorder. For many years back I have been induced to adopt the opinion, that in the secretions furnished by dysentery, we have the evidence of a peculiar form of phlogosis, analogous in many respects to that of croup, and most amenable to those remedies which exert a modifying power over the blood, or what may be termed antiplasmic agents. The efficiency of mercury, where such are indicated, is unquestionable, and it has always been with a view to its alterative action that I have prescribed it. In the management of colitis, two ends have been kept steadily in view, and the satisfactory results obtained confirm me in the course pursued.

First, the removal of all sources of irritation ; and, secondly, alteration, both of the sanguine fluid and the secretory function of the parts involved. To effect these ends, when called to a patient, provided there exists much pain, tender belly, and the dysenteric discharges, I opened a vein and abstracted a quantity of blood sufficient to make a moderate impression upon the circulation, after which, a mild cathartic, such as the Oleum Ricini with xxx. gtt. Tinct. Opii were administered. By these means I got rid of several sources of irritation, as, for instance, a portion of the blood itself (which, when altered in its constitution, may act as any other foreign body,) thus enabling the engorged capillary vessels to empty themselves, and by the removal of all fœcal accumulations and morbid secretions. Having accomplished this, the next step was to place the intestines at perfect rest. By so doing we lower the demand for blood, diminish the depraved functional activity of the parts, and allow the process of repair to go on uninterrupted. In order to effect this, modify the blood and change the secretory action of the vessels. Opium, in combination with calomel, was next administered. Sub. Muriat. Hydrarg. v.—vii. grs. Pul. Opii. vi.—viii. grs. Pul. Ipecac. vii grs. To be divided into four powders and one taken every three hours. If after a time, narcotization occur, the opium should be omitted and the calomel continued, resorting to the former again as soon as the condition of the patient will admit. If the tenesmus continued troublesome, an enema consisting of a little thin starch, to which was added one teaspoonful of Tinct. Opii, generally quieted the bowel. This reflex phenomenon, analogous in many respects to the expulsive efforts of the uterus, is one of the most distressing and harassing attendants of dysentery. In the use of injections to relieve this symptom care should be taken that a very small amount in bulk be employed, as otherwise the stimulus of distension may provoke the bowel to expel it. After the use of the above means, if much tenderness continued over the abdomen, cups were employed with the best results. Leeches would answer equally well, and perhaps in some respects better, but inability to command their use in the country prevented their employment. After the local abstraction of blood, the abdomen was covered by a light, warm, hop poultice, which by its derivative and anodyne effects will be found a very comfortable application to the patient. The drinks employed were barley water, infusion of the ulmus fulvia and benne leaves. This last named article seemed peculiarly appropriate. After the violence of the acute symptoms had passed over, and the bowels still continued to be intolerant of pressure, a fly blister was applied, and after its removal dressed with the ung. hydrarg. fortius. In cases where the discharges were mixed with much blood, the acetas plumbi constituted a valuable addition to the mercurial prescription in doses of three or four grains ; and, indeed, whether the evacuations were of this character or not, if the tongue became red, dry and glazed, the exhibition of lead had a decidedly good effect. For several years I have observed the efficacy of this article in subduing that condition of the mucous membrane of the intestines indicated by the red, dry and glazed tongue, not by any means peculiar to colitis alone, but frequently to be met with in typhoid fever, scarlatina, and as accidental in the course of other febrile affections. In such, it seemed to answer best when administered in solution. Pursuing the above plan, no sooner did the system begin to acknowledge the specific action of the mercurial, than, in the great proportion of cases, the symptoms gave way and convalescence set in. The very mildest action was all that was necessary; and hence, as soon as the least tenderness of the gums or the mercurial fetor was observed, it was either suspended altogether, or, when considered requisite, given only at such intervals as would preserve the impression already made. Occasionally a case would prove rebellious, the stools become more frequent, containing membranous shreds, the belly retracted, the pulse fail, and a disgusting stench emanate from the patient, attended with a confused state of mind. Such a concurrence of symptoms betokened a very serious state of things, and when accompanied by the entire and sudden suspension of pain, likely to prove fatal. The treament adopted in these cases was such as would be calculated to sustain the general system and act upon the diseased surface, arousing, if possible, a degree of vitality sufficiently vigorous to insure its restoration. It is at such a crisis that an appropriate article of nutrition becomes necessary. The one employed was beef essence, and embodying as it does a large amount of nutritive matter in the smallest bulk, and the most unirritating form, we should be unwilling to exchange it for any other article of dietetics in general.

It is at this stage of the disease, as I understand it, that the favorite prescription known as Hope's Mixture is said to prove so curative, and notwithstanding it was employed with strong prejudices in its favor, it proved utterly powerless in my hands; neither did the employment of nitras argenti, so highly recommended in cases of extensive ulceration, produce any reliable results. That which undoubtedly displayed the most decided influence in this stage of the disease, was the following mixture: Ol. Terebinthinæ, ℥iss. Tinct. Catechu, ℥ii. Tinct. Capsici ʒii. Tinct. Opii. gtt. xl.; Mucil. Gum Acaciæ ℥iv. Of this one tablespoonful was given every three hours, and likewise Sulph. Quiniæ grs. ij. every four hours, and where the strength of the patient was not too much exhausted, the belly and hips were immersed in a warm bath. Under this treatment, with an occasional dose of pulv. doveri, and the regular use of beef essence, the most encouraging results were obtained. The average duration of the disease was eleven days. The following table will exhibit the result of ninety-six cases as they occurred indiscriminately:

NINETY-SIX INDISCRIMINATE CASES.

| *Age.* | *Number of Cases.* | *Mortality.* |
|---|---|---|
| Under 5 years, | 19 | 3 |
| " 20 " | 30 | 2 |
| " 50 " | 39 | 3 |
| Over 50 " | 8 | 0 |
| Total, | 96 | 8 |

The administration of even the mildest aperients was not resorted to after the commencement of the disease, unless under certain peculiar circumstances; as, for instance, where the opium effectually blocked up the intestinal discharges for forty-eight hours or more, and even then I much preferred an enema of simple warm water, or warm flax-seed tea, when such had the desired effect; or where the disease attacked gross livers of a full plethoric habit and presenting bilious symptoms; in such, the occasional use of the ol. ricini, to remove the vitiated biliary secretions, was certainly attended with good effects. In reference to the exhibition of mercury, I am aware much prejudice exists, and it is quite certain that many who condemn its employment may have had to deal with colitis under some aspect unfavorable to its use; but in all sincerity I can say, having tested it in those extensive visitations of the disease, and being to thoroughly convinced of its beneficial effects, that to be compelled to encounter a similar epidemic without it would be so feel shorn of half my strength. It does not appear to me that opium exerts, in the management of dysentery, any direct curative effects, but acts much in the same way as the splint in a fractured limb, by keeping the parts at perfect rest while nature performs the cure. This is a very important indication, and hence the utility of combining it with calomel.

The meteorological conditions which seem favorable to the production of colitis, are dull, cloudy weather, accompanied by frequent storms, and an elevated temperature; and of those which favor its declension, none appear more decided than the occurrence of a brisk frost.

In conclusion, it will be perceived, that so far as my observations extended, lime-stone districts have enjoyed a singular security, and to the fact of the inhabitants using water impregnated with lime, has this immunity been ascribed.

## Figures and Tables

**Figure f1:**